# Her2-Targeted Therapy Induces Autophagy in Esophageal Adenocarcinoma Cells

**DOI:** 10.3390/ijms19103069

**Published:** 2018-10-08

**Authors:** Félice A. Janser, Olivia Adams, Vanessa Bütler, Anna M. Schläfli, Bastian Dislich, Christian A. Seiler, Dino Kröll, Rupert Langer, Mario P. Tschan

**Affiliations:** 1 Institute of Pathology, University of Bern, Murtenstrasse 31, 3008 Bern, Switzerland; ariane.janser@pathology.unibe.ch (F.A.J.); olivia.adams@pki.unibe.ch (O.A.); vanessa.buetler@students.unibe.ch (V.B.); anna.schlaefli@pathology.unibe.ch (A.M.S.); bastian.dislich@pathology.unibe.ch (B.D.); 2 Graduate School for Cellular and Biomedical Sciences, University of Bern, Freiestr. 1, 3012 Bern, Switzerland; 3 Department of Visceral Surgery and Medicine, University Hospital Bern, 3010 Bern, Switzerland; christian.a.seiler@insel.ch (C.A.S.); dino.kroell@insel.ch (D.K.)

**Keywords:** autophagy, Her2, Lapatinib, LC3B, p62, OE19, Her2 inhibitor, resistance, esophageal adenocarcinoma

## Abstract

Esophageal adenocarcinoma (EAC) is a highly lethal cancer type with an overall poor survival rate. Twenty to thirty percent of EAC overexpress the human epidermal growth factor receptor 2 (Her2), a transmembrane receptor tyrosine kinase promoting cell growth and proliferation. Patients with Her2 overexpressing breast and gastroesophageal cancer may benefit from Her2 inhibitors. Therapy resistance, however, is well documented. Since autophagy, a lysosome-dependent catabolic process, is implicated in cancer resistance mechanisms, we tested whether autophagy modulation influences Her2 inhibitor sensitivity in EAC. Her2-positive OE19 EAC cells showed an induction in autophagic flux upon treatment with the small molecule Her2 inhibitor Lapatinib. Newly generated Lapatinib-resistant OE19 (OE19 LR) cells showed increased basal autophagic flux compared to parental OE19 (OE19 P) cells. Based on these results, we tested if combining Lapatinib with autophagy inhibitors might be beneficial. OE19 P showed significantly reduced cell viability upon double treatment, while OE19 LR were already sensitive to autophagy inhibition alone. Additionally, Her2 status and autophagy marker expression (LC3B and p62) were investigated in a treatment-naïve EAC patient cohort (*n* = 112) using immunohistochemistry. Here, no significant correlation between Her2 status and expression of LC3B and p62 was found. Our data show that resistance to Her2-directed therapy is associated with a higher basal autophagy level, which is not per se associated with Her2 status. Therefore, we propose that autophagy may contribute to acquired resistance to Her2-targeted therapy in EAC, and that combining Her2 and autophagy inhibition might be beneficial for EAC patients.

## 1. Introduction

Esophageal adenocarcinoma (EAC) is a highly aggressive malignant disease. Locally advanced tumors without distant metastases are treated with peri- or preoperative chemo(radio)therapy and surgery, while patients with metastatic disease can only benefit from palliative chemotherapy [1]. In both clinical scenarios, the high rate of resistance to chemotherapy or radiochemotherapy limits the success of the treatment and thus the survival rate for EAC patients remains low [2]. Therefore, alternative therapeutic options targeting molecular alterations in EAC are in high demand. 

Gene amplifications of the human epidermal growth factor receptor 2 (*Her2*) are reported in 20–30% of EAC cases [3]. It is an oncogenic driver that signals for proliferation and survival. At present, humanized Her2-targeting monoclonal antibody trastuzumab/Herceptin has been approved for the treatment of metastatic Her2-positive gastric cancer and cancers of the gastroesophageal junction [4]. Accordingly, Her2-positive EAC can be treated off-label with Herceptin [5]. A second class of Her2-targeting drugs consists of receptor tyrosine kinase (RTK) small molecule inhibitors such as Lapatinib. Her2-targeting small molecule inhibitors prevent phosphorylation of Her2 by competitively binding to the ATP-binding pocket and in turn prevent oncogenic downstream signaling pathways [6]. Although approved for treatment of Her2-positive metastatic breast cancer, Lapatinib has performed poorly in clinical trials pertaining to gastric cancer, possibly due to heterogeneity, low thresholds of Her2 positivity, and overlapping toxicity profiles with other drugs in combination treatments. Nevertheless, Her2 targeting is still a clinically important therapeutic option which warrants extensive investigation [7].

Macroautophagy (abbreviated here as autophagy) is an evolutionarily conserved lysosome-dependent cellular process. Under basal conditions, autophagy maintains cellular homeostasis by degrading damaged or superfluous organelles and proteins and recycling sugars, amino and fatty acids, as well as nucleotides. Generally, autophagy is a prosurvival mechanism upregulated upon cellular stresses such as nutrient deprivation, metabolic/oxidative stress, or infection [8,9]. In the context of cancer, autophagy has been described as being oncosuppressive as well as oncogenic. In the setting of established tumors, autophagy is thought to maintain cancer stem cells as well as dormant or senescent transformed cancer cells, which is in contrast to its oncosuppresive functions in early stages, where the process maintains normal stem cells. Furthermore, autophagy confers survival advantages to cancer cells under various kinds of antitumoral treatment [10,11].

Her2 signaling and autophagy are linked via the main negative regulator of autophagy, mTORC1, which is downstream of Her2 [12]. In breast cancer, increased autophagic flux has been attributed to resistance to both monoclonal Her2-targeting antibodies and small molecule inhibitors in vitro as well as in vivo [13,14,15]. Moreover, pharmacological or genetic inhibition of autophagy was successful in resensitizing breast cancer cells to Her2-targeted therapy. However, in line with the dual role of autophagy in cancer, contradictory results also have been reported [16]. As intrinsic and acquired resistance to Her2-targeted therapy is well documented in different cancer types, investigating the role of autophagy in Her2-targeted therapeutic response in gastroesophageal cancer could be of clinical relevance. More importantly, routine diagnosis and treatment of Her2-positive EAC cases are realistic in the future [17].

We quantified autophagy regulation upon Her2-targeted therapy using the RTK small molecule inhibitor Lapatinib in OE19 EAC cells. Furthermore, we investigated the potential role of autophagy in acquired resistance against Her2-targeted therapy in EAC in vitro. Moreover, we tested whether the combination treatment of Her2 and autophagy inhibitors is beneficial in treating parental and Lapatinib-resistant OE19 cells. Lastly, in order to investigate a potential intrinsic association between Her2 and autophagy in EAC, we compared the Her2 status with the basal expression of autophagy markers in an EAC patient cohort ex vivo in a treatment-naïve setting.

## 2. Results

### 2.1. Validation of Lapatinib Sensitivity and Her2 Status of OE19 Cells

We used the Her2-positive EAC cell line OE19, as OE19 cells have already been characterized as sensitive to Her2 inhibition. Due to the cell line’s low threshold for Lapatinib, the concentrations used for functional assays in published studies often exceed the IC_50_ value [18,19]. For this purpose, we tested the responsiveness of OE19 to Lapatinib in house and optimized the working concentrations for autophagy and cell viability assays in order to minimize the chance that observed experimental results are attributed to off-target effects. The resazurin-reduction-based alamarBlue^®^ assay was used to determine the relative toxicity of different Lapatinib concentrations on OE19 (Figure S1a). To assess whether the decreased cell viability observed via alamarBlue^®^ assay was due to apoptosis, the luminescence-based Caspase-Glo^®^ 3/7 assay was used. OE19 were treated with the same range of Lapatinib for 24 h and the activity of the caspases 3 and 7 was measured (Figure S1b). Based on these results, we chose 120 nM of Lapatinib as the working concentration. We considered this concentration appropriate for autophagy assays as well as for experiments with combination treatments.

The Her2 status of different EAC cell lines has been described in the literature, and among them, OE19 has been characterized as Her2 positive [19,20]. The methods used to confirm Her2 amplification, however, included array-based comparative genome hybridization (CGH) and varied between studies. Therefore, we validated Her2 status using FISH and immunohistochemistry (IHC) methods used for routine diagnostics of breast and gastroesophageal cancer and which have been tested and validated on EAC patient tissue [21]. In line with the published data, we observed a Her2 amplification in OE19 cells via FISH, with corresponding increased levels of protein observed via IHC score 3+ (Figure S1c,d).

### 2.2. Induction of Autophagic Flux in OE19 Cells upon Lapatinib Treatment

**Figure 1:**
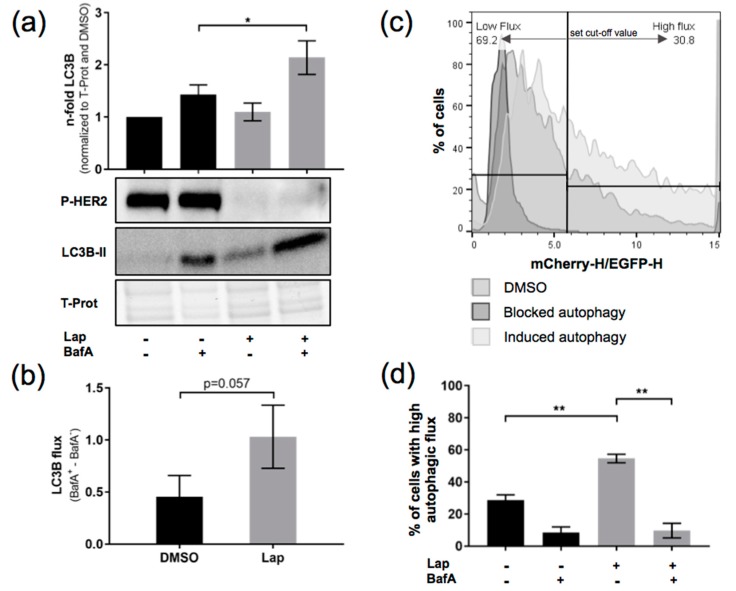
Induction of autophagic flux in OE19 upon Lapatinib treatment. (**a**) LC3B flux was assessed comparing control and BafilomycinA (BafA)-treated (200 nM, 2 h) OE19 upon Lapatinib treatment (120 nM, 24 h). LC3 band intensities were quantified using ImageJ software. Total protein was used as a loading control, and Phospho-Her2 for Lapatinib treatment (*n* = 3). (**b**) LC3B flux was calculated from data in (**a**) as follows: BafA^+^-BafA^−^ values for each condition. (**c**) FACS analysis of mCherry-EGFP-LC3B-expressing OE19 cells upon induction or blockade of autophagy with indication of the chosen cut-off value for high respectively low autophagic flux. (**d**) Quantification of the FACS analysis showing % of cells with high autophagic flux (*n* = 3). Cells were treated as in (**a**). The error bars represent SD, statistical significance was determined by Mann–Whitney U test: * *p* ≤ 0.05, ** *p* ≤ 0.01.

We then set out to find out whether Lapatinib treatment leads to an upregulation of autophagic flux in OE19 cells. The autophagic flux was assessed via LC3B Western blotting. We compared autophagy in blocked and nonblocked conditions of Lapatinib-treated as well as Lapatinib-untreated cells. To block autophagy, BafilomycinA (BafA), a late-stage autophagy inhibitor, was added. As a control for successful Lapatinib treatment, we also checked levels of Phospho-Her2 (Figure 1a). The autophagic flux was calculated (BafA^+^-BafA^−^ conditions) (Figure 1b). In this experiment, we observed an approximative 50% increase in autophagic flux upon Lapatinib treatment. To corroborate this observation, we assessed the autophagic flux using an mCherry-EGFP-LC3 construct and FACS analysis. Upon fusion of the autophagosome with the lysosome, the green fluorescence of the mCherry-EGFP-LC3 construct is quenched due to the lower pH in the autolysosome that leads to a shift to red fluorescence [22]. Using this technique, we observed a significant induction of autophagic flux in OE19 cells upon Lapatinib treatment (Figure 1c,d). BafA-treated OE19 cells were included as a control.

Together, using two LC3-based methods, we could show that Lapatinib treatment leads to an induction of autophagic flux in OE19 cells.

### 2.3. Her2-Inhibition-Resistant OE19 Daughter Cells Show Increased Autophagic Activity Compared to Their Normal Counterparts

**Figure 2:**
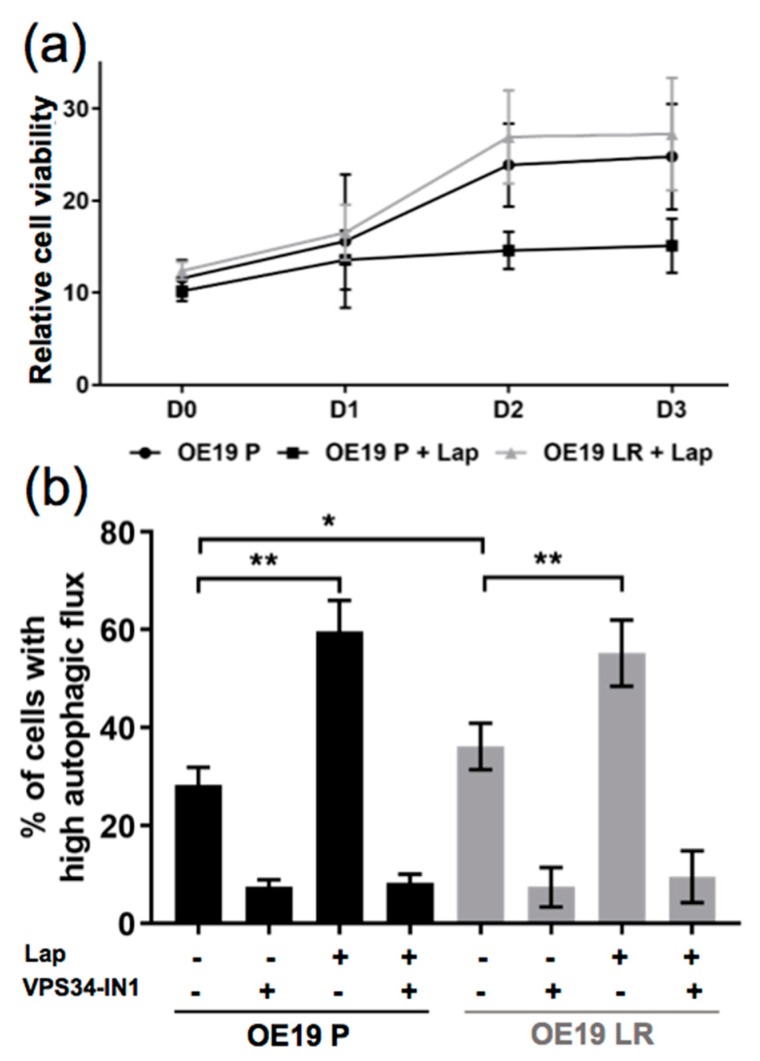
Comparison of the autophagic flux induction in parental (OE19 P) and Lapatinib-resistant (OE19 LR) OE19 cells. (**a**) Relative cell viability assessed by alamarBlue^®^ assay of OE19 P and OE19 LR cells, treated with dimethyl sulfoxide (DMSO) alone or 120nM of Lapatinib (*n* = 3). (**b**) Quantification of FACS analysis comparing OE19 P and OE19 LR transduced with a mCherry-EGFP-LC3B construct (same treatment as in a). As a control, autophagy blocked conditions (addition of 5µM VPS34-IN1) were included, (*n* = 4). The error bars represent SD, statistical significance was determined by Mann–Whitney U test: * *p* ≤ 0.05, ** *p* ≤ 0.01.

In the next step, we generated a Lapatinib-resistant OE19 subline by treating OE19 parental cells (OE19 P) with increasing concentrations of Lapatinib (up to 120 nM) for at least 3 months. Finally, we cultured the cells with 120 nM of Lapatinib to preserve the resistant pool of OE19 cells (OE19 LR). In Figure 2a, an alamarBlue^®^ assay is depicted comparing the relative cell viability of OE19 P and OE19 LR under Lapatinib treatment.

We used the mCherry-EGFP-LC3 construct and FACS analysis to compare the autophagic flux induction in these two cell lines upon Lapatinib treatment. We observed a significantly higher basal autophagic flux in OE19 LR compared to OE19 P cells. Moreover, Lapatinib treatment resulted in a significant induction of flux in both lines (Figure 2b). In addition, we used a VPS34 kinase inhibitor (VPS34-IN1), a novel, early stage autophagy inhibitor [23]. Using this inhibitor, both cell lines responded by showing significantly decreased basal autophagy levels. The combination of VPS34-IN1 with Lapatinib led to a reduction of autophagy back to basal autophagy activity (Figure 2b). Taken together, we observed that OE19 LR cells show significantly increased basal autophagy. Moreover, Lapatinib-induced autophagy can be blocked by a specific autophagy inhibitor inactivating VPS34 kinase.

### 2.4. Blocking Autophagy in Combination with Her2 Inhibition Significantly Reduces OE19 Cell Viability and Colony Formation

Since we observed that OE19 LR showed significantly higher basal autophagy compared to OE19 P cells, we tested whether combining Lapatinib with autophagy inhibitors would resensitize the resistant cells. For these experiments, we used the previously described VPS34 inhibitor as well as chloroquine (CQ), which is used in the clinic not only for malaria treatment but also for cancer therapy, partly in combination regimens with standard cytotoxic chemotherapies [24].

**Figure 3:**
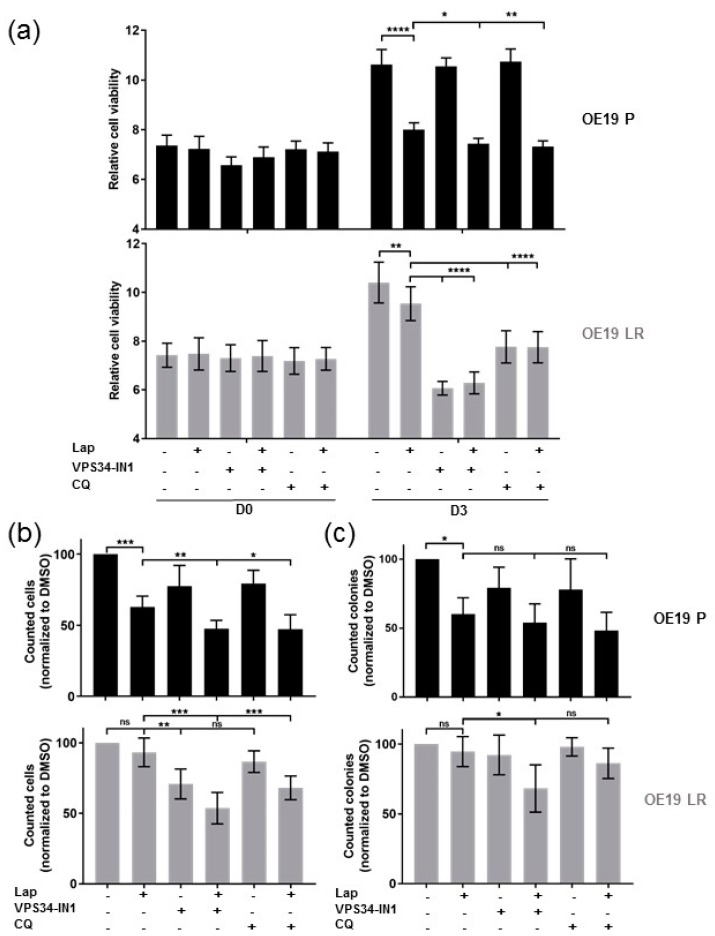
Combined Her2 and autophagy inhibitor treatment (**a**) Relative cell viability of OE19 cells treated with Lapatinib (120 nM) and/or autophagy inhibition either VPS34 inhibitor (VPS34-IN1) (5 µM) or chloroquine (CQ) (25 µM) at days 0 and 3 of the alamarBlue^®^ experiment (*n* ≥ 3). (**b**) Cell counts of OE19 P and OE19 LR cells treated as in (**a**) but for 48 h. Values were normalized to DMSO control treated cells (*n* = 7) (**c**) Colony numbers after treatment as described in (**b**), reseeded (2500 cells/well) in six-well plates and incubation for 14 days without treatment (*n* = 5). The error bars represent SD, statistical significance was determined by Mann–Whitney U test: ns = non significant *p* > 0.05, * *p* ≤ 0.05, ** *p* ≤ 0.01, *** *p* ≤ 0.001, **** *p* ≤ 0.0001.

In the parental OE19 P cells, inhibiting autophagy alone (VPS34-IN1 or CQ) showed no significant effect on the relative cell viability. However, the combination treatments showed a significant further reduction in cell viability compared to the single Lapatinib treatment. Differently, the effect of the autophagy inhibitor treatments alone as well as the combination with Lapatinib showed comparably significant effects on the Lapatinib-resistant OE19 LR cells (Figure 3a and Figure S2).

In line with the cell viability assays, autophagy inhibition showed no effect in the parental cell line, while the double treatment with Lapatinib reduced the cell numbers significantly compared to Lapatinib treatment alone. In OE19 LR, inhibition of autophagy with the more specific inhibitor (VPS34-IN1) resulted in a significant reduction in cell numbers, whereas this effect was not seen using chloroquine. Combining Lapatinib with either autophagy inhibitor resulted in a significant reduction of viable OE19 LR cells (Figure 3b).

Additionally, we performed colony formation assays to address tumor cell recovery. After 48 h of treatment, cells were reseeded in very small numbers (identical for all conditions), and after an incubation of 14 days without treatment, the colonies were counted. Treating Lapatinib-sensitive OE19 P with Lapatinib alone or in combination with autophagy inhibitors resulted in a significant reduction in colonies compared to the control conditions. Interestingly, treatment of OE19 LR with Lapatinib and the VPS34 inhibitor caused a significant reduction in colony numbers, while all other conditions did not show any significant reduction (Figure 3c and Figure S3).

In summary, our results indicate that blocking autophagy in combination with Lapatinib in OE19 EAC cells showed significantly better treatment effects than Lapatinib treatment alone. Moreover, combining autophagy and Her2 inhibition allowed the successful treatment of Lapatinib-resistant EAC cells. Importantly, likely based on the increased basal autophagy of OE19 LR cells, these cells respond to autophagy inhibition alone under certain conditions.

### 2.5. Assessment of Her2 Status and Autophagy Marker Levels in a Treatment-Naïve EAC Patient Cohort

**Figure 4:**
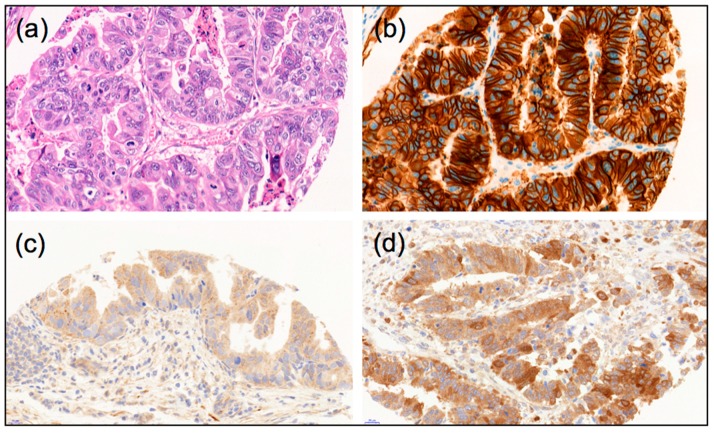
Examples of morphology, autophagy markers and Her2 immunohistological stainings of esophageal adenocarcinomas: (**a**) Hematoxylin-Eosin stain; (**b**) Example of strong Her2-positive staining; (**c**) Example of LC3B high dot-like staining; (**d**) Example of p62 high cytoplasmic/dot like staining, (40× magnification each).

Our in vitro results suggest a role of autophagy in response to Her2-targeted therapy and prompted us to investigate the relationship between basal autophagy under steady-state conditions and Her2 status in primary resected human EAC tumor samples. The rationale for this assessment was that amplified Her2 signaling results in increased mTORC1 signaling, leading to autophagy suppression. To this end, we determined the Her2 status of a well-characterized treatment-naïve EAC cohort via IHC [25]. The IHC scores for Her2 were compared to previously published scores for LC3B and p62 [25]. Examples of LC3B and p62 IHC stainings are shown in Figure 4.

Ninety-four tumors were Her2 negative and 18 tumors were Her2 positive. Her2-positive tumors were more frequently well and moderately differentiated (*p* = 0.016), but there were no significant correlations with other pathological features such as pT or pN category. There was also no correlation between Her2 status and the expression of the autophagy markers LC3B or p62 (*p* = 0.6 and *p* = 0.8, respectively). The correlation data is presented in Table 1 for LC3B and Table 2 for p62.

## 3. Discussion

We observed induction of autophagic flux upon Lapatinib treatment in OE19 EAC cells using two different LC3B-dependent methods for assessing autophagy. These observations are in line with studies showing autophagy induction upon Lapatinib treatment in breast cancer [13,26]. We further observed that OE19 cells that acquired resistance to Lapatinib exhibit a higher basal autophagy and that the effect of autophagy induction upon Lapatinib treatment is less pronounced in these cells compared to the normal counterpart. These findings indicate that autophagy is involved in resistance formation against Lapatinib in EAC cells. Therefore, modulation of autophagy could potentially be used as a mechanistic tool to overcome resistance to anticancer treatment, which represents the most relevant clinical challenge in tumor therapy. EAC autophagy has already been reported to enhance the efficacy of cancer therapies, and clinical trials are ongoing using hydroxychloroquine (HCQ), an FDA-approved derivative of chloroquine used for malaria treatment, as an autophagy inhibitor in cancer treatment. One important finding of these studies is that HCQ can be combined with other drugs such as chemotherapies [24]. Protective autophagy and its role in the formation of resistance to Her2-targeted treatment has been discussed for Her2-positive breast cancer in vitro and in vivo [15,16]. Moreover, autophagy has been described as a resistance mechanism against conventional chemotherapy in EAC cells [27].

Based on the abovementioned observations, we investigated the effect of Lapatinib in combination with autophagy inhibitors on OE19 cell lines. We observed a reduction in relative cell viability, cell amounts, and colony formation capacity upon these combination treatments. Using the alamarBlue^®^ assay, we observed a highly significant reduction of relative cell viability upon treatment with autophagy inhibitors alone in the resistant cells, whereas these had almost no effect on the parental cells. Looking at the cell counts of viable cells after treatment, a slightly different picture was observed. In the resistant cells, the combination treatments were more effective than autophagy inhibition alone. However, we observed in these cells already a significant effect (*p* = 0.0064) of VPS34-IN1 alone compared to the Lapatinib treatment, whereas the autophagy inhibition alone had no significant effect on the parental cells. The only significant reduction in colony formation capacity was seen for the resistant cells upon combination of Lapatinib and VPS34-IN1. Taken together, these results indicate that the resistant population of OE19 cells in general recovers better from treatment. Compared to the parental cells, the resistant cells were generally more sensitive to the autophagy inhibitors. Especially, the treatment of the resistant cells with VPS34-IN1 alone and in combination with Lapatinib showed intriguing effects.

Our observations upon the combination of Lapatinib and autophagy inhibitors are in line with other studies carried out in breast and bladder cancer models [26,28]. Both of these studies based their conclusions mostly on results using chloroquine as an autophagy inhibitor [24]. However, it has been shown that chloroquine does not only impair autophagic flux but also leads to multiple cellular alterations, such as the disorganization of the Golgi and endolysosomal networks [29]. Thus, we included in our study a promising, more specific autophagy inhibitor VPS34-IN1. This inhibitor is highly specific for the lipid kinase VPS34, which catalyzes the phosphorylation of phosphatidylinotisol to phosphatidylinositol 3-phosphate required for formation of new autophagosomes [23]. This early stage autophagy inhibitor is also in preclinical development [30].

In clinical practice, the treatment of gastric and gastroesophageal adenocarcinoma with Lapatinib alone seems to be more challenging compared to its application in breast cancer. Particularly, it seems that it is only suitable for a subset of patients [31,32]. We tested whether Her2 status itself may already influence autophagy under basal conditions. In an ex vivo approach, we correlated the Her2 status of primary resected treatment-naïve EAC tumor samples with the expression of LC3B and p62. We found no primary correlation between the Her2 status and these autophagy-related proteins that are widely used for approximately monitoring autophagy in tissue samples using detection by immunohistochemistry. These results indicate that some of the tumors exhibit high while others exhibit low levels of basal autophagy independently of the Her2 status. Regarding our in vitro results, one could suggest that there are tumors that exhibit high basal autophagy, which would be Lapatinib resistant and would not respond to the treatment. Unfortunately, we could not analyze tumor tissue prior to Her2-directed treatment and compare the results of the expression analysis with response, since gastroesophageal adenocarcinoma patients are currently only treated in a metastatic setting outside of clinical trials. Clinically well-annotated case collections, such as the cohort of primary resected tumors that we used for this study for studying a potential basal correlation between Her2 status and the expression of autophagy-related proteins, are not available yet.

Taken together, our study could serve as the basis for future investigations addressing resistance mechanisms to Her2-targeted treatment in EAC. Our results indicate that autophagy could be involved in resistance formation against Lapatinib treatment and that the combination treatment of Her2-targeted therapy and autophagy inhibition represents a promising treatment strategy for Her2-positive EAC patients.

## 4. Materials and Methods

### 4.1. Drugs and Inhibitors

Lapatinib (Lap) (Selleckchem, LubioScience Luzern, Switzerland, S2111) was reconstituted in dimethyl sulfoxide (DMSO) and stock solutions were stored at −80 °C. Inhibitors were diluted freshly in standard growth medium before each experiment. Chloroquine (CQ) was purchased from Sigma-Aldrich (Buchs, Switzerland, C6628) and was dissolved in distilled water and stored at −20 °C. Bafilomycin A1 (BafA) was purchased from LC Laboratories (Woburn, MA, USA, B-1080). It was dissolved in DMSO and stored at −20 °C. The VPS34 inhibitor (VPS34-IN1) was purchased from Selleckhchem (1383716-33-3), diluted in DMSO, and stored at −20 °C.

### 4.2. Cell Culture and Generation of Resistant OE19 Cells

The human EAC cancer cell line OE19 from the Public Health England Culture Collections was purchased from Sigma-Aldrich, Buchs, Switzerland. The cells were cultured in RPMI-1640 (Sigma-Aldrich, R8758) supplemented with 10% fetal bovine serum (Sigma-Aldrich, F7524) and 1% penicillin streptomycin (Sigma-Aldrich, P4333). Cells were kept in a humidified incubator with 5% CO_2_ at 37 °C. The human embryonic kidney cell line 293T used for lentivirus production was cultured and maintained in DMEM (Sigma-Aldrich, D6046) supplemented with 5% FBS, 1% penicillin/streptomycin, and 1% HEPES (Sigma-Aldrich, H3375) in a humidified incubator with 7.5% CO_2_ at 37 °C. To generate a pool of OE19 cells showing secondary resistance to Lapatinib, OE19 P were exposed to increasing concentrations of Lapatinib for at least 5 months. Initially, the cells were exposed to 20 nM of Lapatinib, followed by 40, 60, 80, 100, and finally 120 nM of Lapatinib. The dosage of the drug was increased after at least 4 weeks of treatment or after the cells were adapted to the drug. In between drug administration, the cells were cultured in normal growth medium over the weekend. The generated pool of resistant cells was maintained in medium with 120 nM of Lapatinib and was cultured before the experiments in normal growth medium without the drug for 2 days.

### 4.3. Western Blotting

OE19 cells were washed in phosphate buffered saline (PBS) and lysed in urea buffer (8 M urea, 0.5% triton X) containing protease inhibitor and PhosSTOP (both: Roche Diagnostics, Rotkreuz, Switzerland). Samples were sonicated, centrifuged (13,000 rcf for 30 min), and the protein concentration was determined via Bradford assay (BioRad, Cressier, Switzerland). Twenty micrograms of protein were denatured in a self-made sample buffer with β-mercaptoethanol (Sigma-Aldrich, M-7522) at 95 °C for 5 min. Samples were loaded on a 4–20% stain-free precast gel (BioRad) and transferred onto a polyvinylidene difluoride membrane using the Trans-Blot^®^ Turbo™ Transfer system (BioRad). Total protein was visualized as loading control using the ChemiDoc™ MP system (BioRad). The following primary antibodies were used: Anti-LC3B from Novus Biologicals (rabbit polyclonal, #NB600-1384, LuBioScience, Luzern, Switzerland) and the anti-Phospho-Her2 from Cell Signaling (rabbit monoclonal, clone (Tyr1221/1222) (6B12), #2243P, Leiden, The Netherlands). The membranes were blocked in 5% BSA/tris-buffered saline (TBS) for LC3B and 5% milk/TBS for Phospho-Her2. Working solutions of the primary antibodies were prepared with a final dilution of 1:1000 in 5% milk/TBS with 0.1% Tween (Sigma Aldrich, P9416) (TBS-T) for LC3B and 5% BSA/TBS-T for Phospho-Her2. Membranes were incubated overnight at 4 °C. DyLight^®^650 conjugated goat anti-rabbit for LC3B (LabForce, Muttenz, Switzerland) and respectively HRP coupled goat anti-rabbit (CellSignaling Technology, Danvers, MA, USA) for Phospho-Her2 were used as secondary antibodies and diluted 1:1000 or 1:10,000 in 5% milk/TBS-T. Membranes were incubated for 1 h at room temperature. Proteins of interest were visualized using the ChemiDoc™ MP system (BioRad). Bands were quantified using ImageJ software (1.64r; NIH, Bethesda, MD, USA).

### 4.4. Autophagic Flux Analysis by Flow Cytometry

The mCherry-EGFP-LC3-expressing lentiviral vector was kindly provided by Dr. Maria S. Soengas (Molecular Pathology Program, Centro Nacional de Investigaciones Oncologicas, Madrid, Spain). The vector contains a puromycin-resistant gene for selection of transduced mammalian cells. Lentivirus production and cancer cell transduction were done as described in [33,34]. Transduced OE19 cells were selected with puromycin (1.5 mg/mL) for 4 days. The mCherry-EGFP-LC3B-expressing cells were seeded in 24-well flat-bottom plates at a density of 120,000 cells/well and on the next day treated as indicated for 24 h. The FACS experiments were performed on a FACS BD LSR II (BD Biosciences, San Jose, CA, USA) using BD FACSDiva software. The data was analyzed using FlowJo software, v10 (Ashland, OR, USA). To estimate the percentage of cells with high autophagic flux, a gate based on the DMSO control was set as described elsewhere [22].

### 4.5. alamarBlue^®^ Assay

The relative cell viability of OE19 cells after treatment was assessed using the alamarBlue^®^ assay (ThermoFisher Scientific, Reinach, Switzerland, DAL1100) according to the manufacturer’s instructions. The alamarBlue^®^ reagent consists of a redox indicator containing the dye resazurin, appearing blue in its oxidized and red in its reduced form. Metabolically active cells catalyze the reduction of the dye, resulting in a colorimetric change. The cells were plated in 96-well flat-bottom plates at a density of 7000 cells/well and allowed to adhere overnight. Afterwards, the treatment was applied and the mixture was incubated with the alamarBlue^®^ reagent for 2 h prior to measuring absorbance at 570 and 600 nm for each indicated time point. A DMSO control was used as a reference. The reduction of the alamarBlue^®^ reagent was calculated and represented as relative cell viability.

### 4.6. Colony Forming Assay and Cell Counting

To assess the ability of cells to recover from treatment, they were treated for 48 h before trypsinization. Then, the viable cells were counted using trypan blue with an automated cell counter (Countess II FL, ThermoFischer Scinetific, Reinach, Switzerland). Afterwards, the cells were replated at equal numbers (2500 cells) in six-well plates (each condition in triplicate). After 14 days in normal growth medium, the colonies were fixed and stained with 0.05% crystal violet in 30% ethanol. Colony numbers were counted with GelCount™ (Oxford Optronix, Milton Park, UK).

### 4.7. Caspase-Glo^®^ 3/7 Assay

The Caspase-Glo^®^ 3/7 assay (Promega, Dübendorf, Switzerland, G8091) was used to determine the activity of the caspases 3 and 7. Cells were seeded in white-walled 96-well flat-bottom plates and allowed to adhere overnight. Afterwards, the cells were treated with Lapatinib at indicated concentrations for 24 h. The Caspase-Glo^®^ 3/7 reagent was mixed according to the manufacturer’s instructions, added to the wells in a 2:3 ratio, and the mixture was incubated for 1 h at room temperature in the dark. The plate was shaken for 3 s at an amplitude of 1 mm before resulting luminescent signals, proportional to caspase activity, was measured using a luminometer (Tecan Infinite^®^200 PRO, Tecan, Männedorf, Switzerland).

### 4.8. Statistical Analysis

All data of the in vitro experiments were expressed as mean ± SD To compare differences between groups, nonparametric Mann–Whitney U tests were applied using the Prism software. A *p*-value < 0.05 was considered statistically significant.

For correlative statistical analysis of the immunohistochemical stainings, IBM SPPS Statistics 24 (IBM Corporation, Armonk, NY, USA) was used. Group comparisons were performed using crosstabs, χ-squared tests, and Fisher’s exact tests. The significance level was set for a *p*-value of <0.05.

### 4.9. Patients and Tissue Samples

Formalin fixed, paraffin embedded (FFPE) tumor tissue from 112 esophageal adenocarcinoma patients was used to generate a tissue microarray (TMA). Tumors of all stages were included. The tumors were all primary resected without prior neoadjuvant chemotherapy, radiochemotherapy, or Her2-directed treatment, respectively. Details of the case collection have been described before [25]. The use of human archival pathological tissue for TMA-based studies was approved by the local ethics committee (Kantonale Ethikkommission Bern, Switzerland, 200/14; approved 17 February 2015).

### 4.10. Immunohistochemistry for FFPE Tumor Tissue

Immunohistochemical stainings were performed on 4-µm sections of the tissue microarray using automatic immunostainers. IHC for Her2 (clone 4B5, Ventana Roche, Rotkreuz, Switzerland) was performed using the Benchmark Ultra immunostainer (Ventana Roche), undiluted, with EDTA antigen retrieval for 36 min. IHC for LC3B and p62 was performed using the Leica Bond RX immunostainer (Leica Biosystems, Muttenz, Switzerland) as described in detail before [35]: briefly, the anti-LC3B antibody (Novus Biologicals #NB600-1384) was diluted 1:4000 and incubated at 95 °C for 30 min. The anti-p62/SQSTM1 antibody (MBL rabbit polyclonal, #PM0045, LabForce, Nunningen, Switzerland) was diluted 1:9000 and incubated at 95 °C for 30 min. The Bond Polymer Refine Detection kit (Leica Biosystems, DS9800) and the Ultra view DAB detection kit (Ventana Roche) were used for visualization according to the instructions of the manufacturer. Scoring of immunohistochemical staining patterns for LC3B and p62 and categorization into high and low expression were performed as described in detail before for this patient cohort [25]. Scoring was performed by one experienced pathologist (RL).

### 4.11. Evaluation of Her2 Status and Classification of Her2-Positive Tumors

Evaluation of Her2 was carried out according to published guidelines by RL [36]. The classification of tumors into Her2 negative (score 0 and 1) and Her2 positive (score 2 and 3) was performed as described elsewhere [37].

## 5. Conclusions

To our knowledge, we are the first to show induction of autophagic flux upon Lapatinib treatment in esophageal adenocarcinoma cells. Furthermore, we observed an elevated basal autophagic flux as well as a more pronounced upregulation of autophagic flux upon Lapatinib treatment in Lapatinib-resistant EAC cells. By combining Lapatinib with autophagy inhibition, we observed a significant effect on cell viability in parental and resistant cells, whereby the effect was more pronounced in resistant cells. In addition, we assessed the regeneration capacity of the tumor cells after single and double treatments using a colony formation assay. The parental cells did not show a significant reduction of colonies in response to the autophagy inhibitors alone but responded to Lapatinib treatment alone or in combination with autophagy inhibitors. Importantly, Lapatinib-resistant EAC cells showed a significant reduction in colony formation capacity only when treated with VPS34-IN1 and Lapatinib. As for the ex vivo data, we observed no correlation between the autophagy markers LC3B and p62 and the Her2 status of EAC cases in a treatment-naïve setting. Currently, we do not have access to tissue samples of EAC patients who were treated with Lapatinib and can therefore not analyze our results in the context of treatment resistance. Nevertheless, we speculate that autophagy marker levels could be associated with treatment resistance respectively with responsiveness.

Overall, we showed a beneficial effect of the combination treatment with Lapatinib and autophagy inhibition on Her2-inhibitor sensitive as well as resistant OE19 EAC cells. Reviewing our results, we propose that autophagy may contribute to acquired resistance to Her2-targeted therapy in EAC, and that combining Her2 and early autophagy inhibition might be a future treatment option for EAC patients.

## Figures and Tables

**Table 1:** Results of the LC3B scoring in correlation with the Her2 status.

		LC3B		Total
		Low	High	
**Her2**	**negative**	50	44	94
	**positive**	8	10	18
total		58	54	112

**Table 2:** Results of the p62 scoring in correlation with the Her2 status.

		p62		Total
		Low	High	
**Her2**	**negative**	21	73	94
	**positive**	3	15	18
total		24	88	112

## Supplementary Materials

Supplementary materials can be found at http://www.mdpi.com/1422-0067/19/10/3069/s1.
