# Supplementary figures and images for: Her2-Targeted Therapy Induces Autophagy in Esophageal Adenocarcinoma Cells
Source: Int J Mol Sci. 2018 Oct 8;19(10):3069. doi: 10.3390/ijms19103069 (PMC6213363; doi:10.3390/ijms19103069)

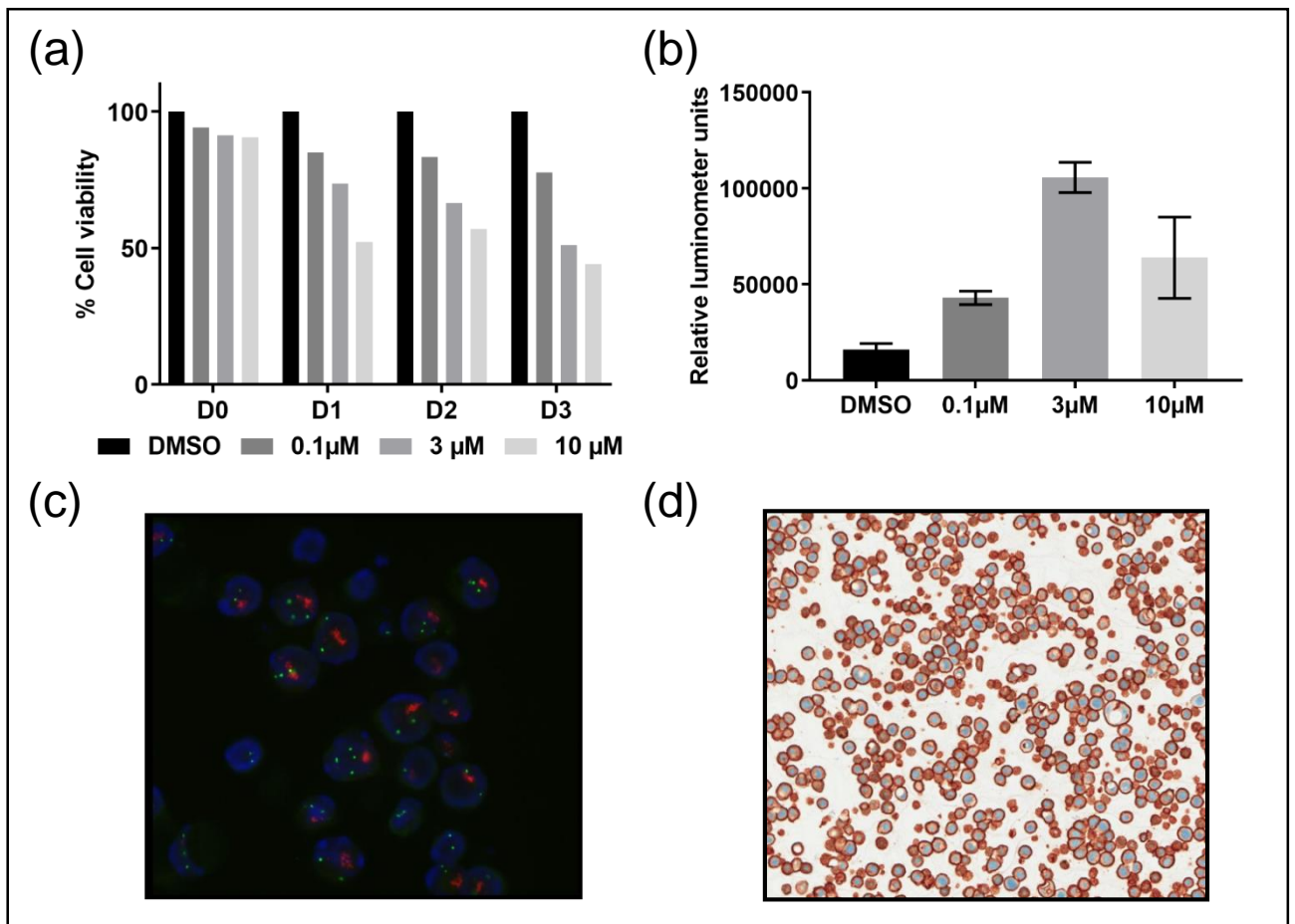

**Figure S1.** Validation of Lapatinib sensitivity and Her2 status of OE19 cells.

Supplement: Supplementary file 1 [file ijms-19-03069-s001.zip › Supplementary Figure 1.pdf]

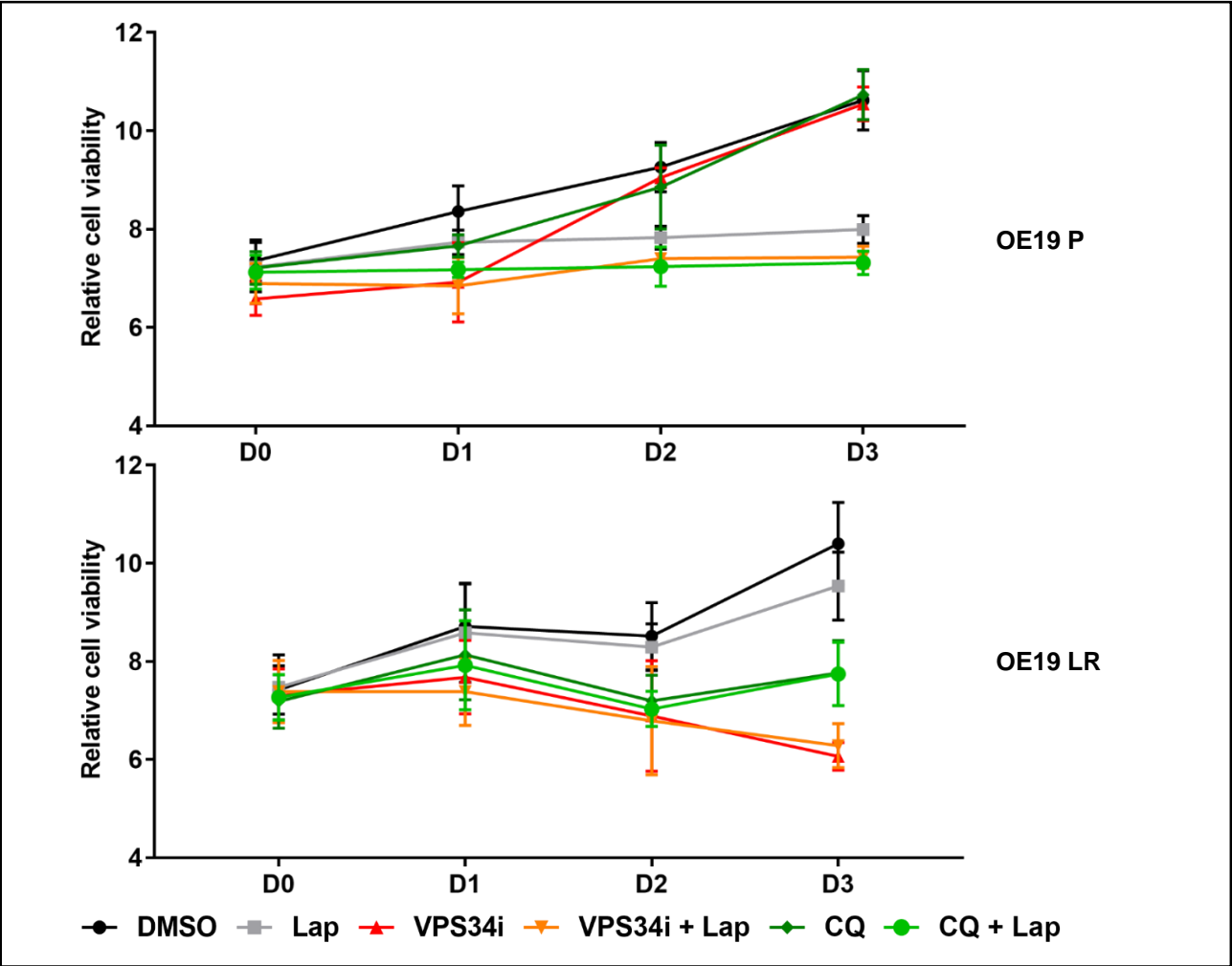

**Figure S2.** Complete data (D0-D3) of the alamarBlue® assays shown in Figure 3 (a).

Supplement: Supplementary file 1 [file ijms-19-03069-s001.zip › Supplementary Figure 2.pdf]

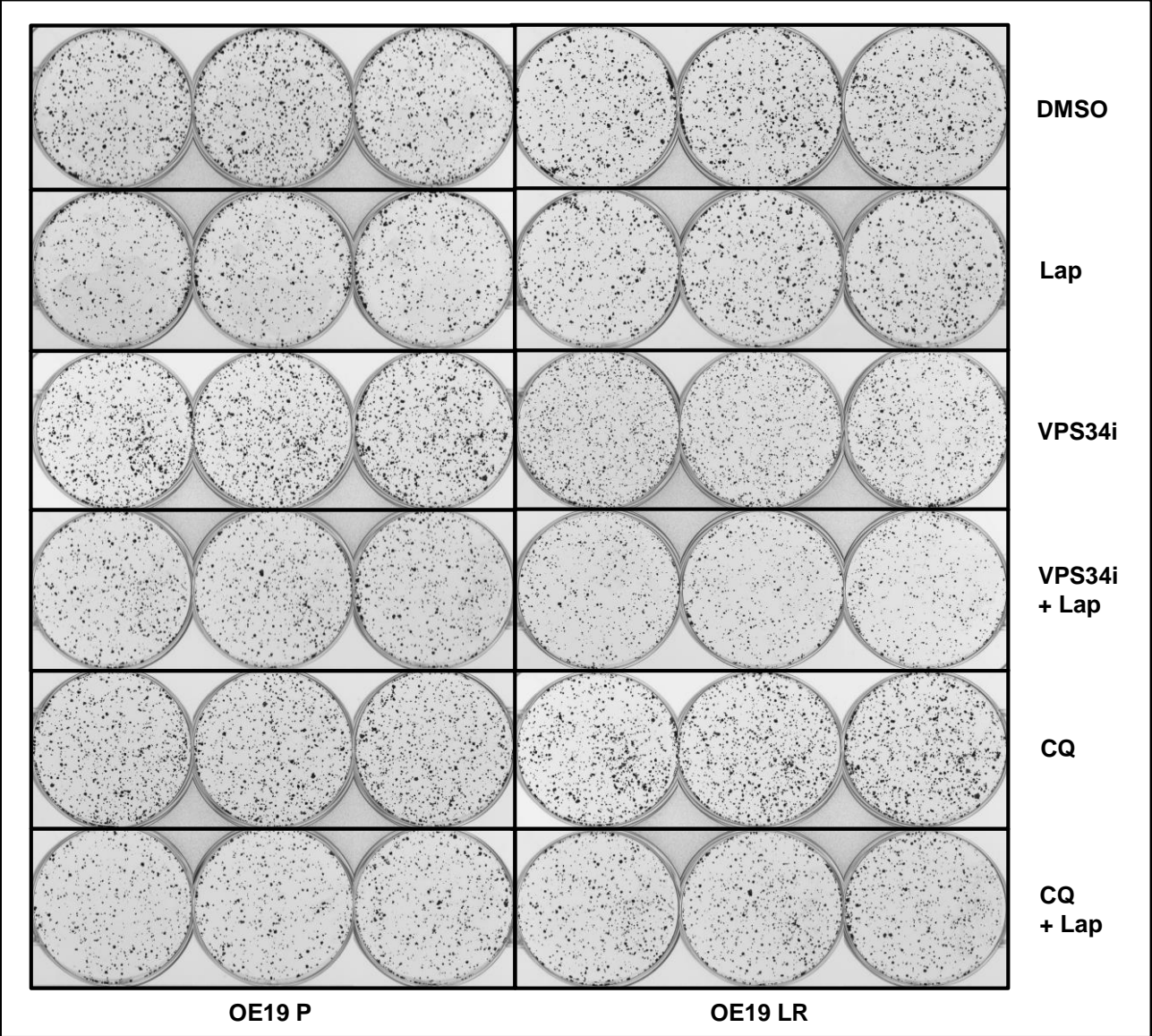

**Figure S3.** Representative pictures of the colony formation assays shown in Figure 3 (c).

Supplement: Supplementary file 1 [file ijms-19-03069-s001.zip › Supplementary Figure 3.pdf]
